# Examining the 10-Year Rebuilding Dilemma for U.S. Fish Stocks

**DOI:** 10.1371/journal.pone.0112232

**Published:** 2014-11-06

**Authors:** Wesley S. Patrick, Jason Cope

**Affiliations:** 1 Office of Sustainable Fisheries, National Marine Fisheries Service, Silver Spring, Maryland, United States of America; 2 Northwest Fisheries Science Center, National Marine Fisheries Service, Seattle, Washington, United States of America; Technical University of Denmark, Denmark

## Abstract

Worldwide, fishery managers strive to maintain fish stocks at or above levels that produce maximum sustainable yields, and to rebuild overexploited stocks that can no longer support such yields. In the United States, rebuilding overexploited stocks is a contentious issue, where most stocks are mandated to rebuild in as short a time as possible, and in a time period not to exceed 10 years. Opponents of such mandates and related guidance argue that rebuilding requirements are arbitrary, and create discontinuities in the time and fishing effort allowed for stocks to rebuild due to differences in productivity. Proponents, however, highlight how these mandates and guidance were needed to curtail the continued overexploitation of these stocks by setting firm deadlines on rebuilding. Here we evaluate the statements made by opponents and proponents of the 10-year rebuilding mandate and related guidance to determine whether such points are technically accurate using a simple population dynamics model and a database of U.S. fish stocks to parameterize the model. We also offer solutions to many of the issues surrounding this mandate and its implementation by recommending some fishing mortality based frameworks, which meet the intent of the 10-year rebuilding requirement while also providing more flexibility.

## Introduction

Managing marine fisheries for sustainable yield has been a goal of fishery managers for centuries [1], [2], [3], yet today many of the world's fisheries still suffer from overexploitation [4], [5], [6]. The various consequences of depleting a fishery resource include economic (e.g., sub-optimal yields), social (e.g., reduced workforce), and ecological (e.g., reductions in the resiliency of the marine ecosystem) impacts [7], [8]. Rebuilding overexploited fisheries to sustainable levels of catch can take several years to decades, depending on the productivity of the stocks (which may change due to environmental and biological conditions), the history and degree of depletion, and fishing mortality rate within those fisheries [9], [10]. Thus, fishery managers must consider the ecological, social, and economic trade-offs of rebuilding immediately versus rebuilding more slowly over time.

In the United States, federally managed marine fisheries are mandated to rebuild the biomass (*B*) of overfished stocks (i.e., often defined as *B* <½ *B_msy_*) to levels that support maximum sustainable yield (*B_msy_*) in as short a time as possible, accounting for the status and biology of the stock, the needs of the fishing communities, recommendations by international organizations in which the U.S. participates, and the interactions within the marine ecosystem (Section 304(e)(4) of the Magnuson-Stevens Fishery Conservation and Management Act (MSA), as amended by the Sustainable Fisheries Act (SFA) (11). Furthermore, overfished stocks must be rebuilt within 10 years, except in cases where the life history characteristics of the stock, environmental conditions or management measures under an international agreement dictate otherwise [11].

The legislative history behind the 10-year requirement was not documented by Congress; however, Safina et al. [9] asserts that the 10-year requirement to rebuild was the result of several population dynamics experts stating, during the drafting of the SFA in 1996, that many overfished stocks were capable of rebuilding to maximum sustainable yield within 5 years if there was a moratorium on fishing. The drafters of the SFA then looked at balancing the short- and long-term trade-offs, and decided that 10 years (twice the time needed for most stocks to rebuild) was a reasonable timeframe to ensure stocks rebuild in a timely manner while accounting for socio-economic impacts [9].

In 1998, NOAA's National Marine Fisheries Service (NMFS; the federal agency responsible for managing marine fisheries) developed national guidance on rebuilding overfished stocks to operationalize the 1996 SFA mandate to rebuild in as short a time as possible [12]. The guidance provided managers with a framework to determine the targeted time to rebuild (*T_target_*) by specifying a minimum (or quickest) time for rebuilding a stock (*T_min_*) and a maximum time allowable for rebuilding a stock (*T_max_*). *T_target_* is then set somewhere between *T_min_* and *T_max_* based on an analysis of the factors listed previously (MSA Section 304(e)(4)). *T_min_* is defined as the expected amount of time a stock needs to rebuild to *B_msy_* in the absence of fishing mortality. In this context, the term “expected” means a 50 percent probability of attaining the *B_msy_* given inherent uncertainty in projecting biomass. For stocks that have a *T_min_* of 10 years or less, the *T_max_* cannot exceed 10 years. If *T_min_* exceeds 10 years, then *T_max_* is calculated as *T_min_* plus one generation time for that stock, where “generation time” is defined as the average age of spawning individuals within a population [13]. Once *T_target_* has been chosen by fishery managers based on their 304(e)(4) analysis, a rebuilding plan is developed which often specifies a constant rebuilding fishing mortality rate (*F_rebuild_*) that is some percentage of the rate associated with achieving maximum sustainable yield (*F_msy_*) [14].

Since the implementation of the SFA in 1996 and NMFS's 1998 guidance, these rebuilding requirements have been both praised and criticized publicly [12], [15]. More recently, issues with rebuilding led Congress to require that NMFS fund a study by the National Academy of Science's National Research Council (NRC) to evaluate the effectiveness of the current rebuilding requirements [8]. In general, proponents believe the requirements were needed to curtail practices of inaction by managers to prevent overfishing (i.e., *F*> *F_msy_*) on rebuilding stocks and laissez-faire attempts to meet rebuilding targets [9], [16], [17]. For example, both Rosenberg et al. [18] and Milazzo [14] found overfishing occurring in 40 to 45% of the stocks under rebuilding plans, and in some cases overfishing had persisted for more than 5 years. Furthermore, at least 22% of plans had reset rebuilding deadlines back to year 1 when the plans were revised, instead of using the existing time frame. This practice allowed managers to extend the rebuilding time frames well beyond the plain language of the SFA [18].

However, opponents note that the 10-year requirement has limited the way in which managers can consider the socio-economic impacts of rebuilding plans. For example, stocks unlucky enough to have a *T_min_* of 10 years would be subject to a 10-year moratorium [8], [12], [15]. Such a moratorium could wreak havoc on the infrastructure and markets of the fishing industry if the stock makes up a key component of the fishery [19], [20], or could severely limit fishing opportunities for other stocks in the fishery due to bycatch issues [21]. Such a scenario is unlikely when *T_min_*>10 years. The discontinuity in the treatment of *T_max_* is also viewed as unfair [8]. For example, if a stock could be rebuilt in 11 years in the absence of fishing pressure (instead of 10), the stock would not be subject to a moratorium and could have a longer *T_max_* (i.e., 11 years plus one generation time of the stock). Opponents also point to discontinuities in the guidance that allows stocks that can rebuild in less than 10 years (using the *T_min_* plus one generation calculation) to still have a 10-year *T_max_*, because of the SFA mandate that specifies that stocks should rebuild in as short a time as possible [12], [15].

We evaluate these statements made by proponents and opponents of the 10-year rebuilding mandate and guidance to determine whether such points are technically accurate, and offer a resolution to some of the issues surrounding this mandate and its implementation. Lastly, it is worth noting, that many of the statements evaluated below are based on the findings of Safina et al. [9]. Thus, for comparison sake, our modelling exercises replicate that of Safina et al. [9], rather than using more sophisticated modelling techniques that are more commonly used in fisheries management.

## Can Most Stocks in the United States Rebuild in 5 Years under Moratorium Conditions?

As mentioned earlier, Safina et al. [9] is the primary source of information that explains why the 10-year rebuilding timeframe was chosen (i.e., twice the time needed to rebuild most stocks). In that article, the authors relied on a Graham-Schaefer model to describe why most stocks can rebuild within 5 years. The model estimated rebuilding times (*t*) based on the intrinsic rate of population increase (*r*), fishing mortality (*F*) relative to the rate associated with MSY (*F_msy_*, thus *F_ratio_ = F/F_msy_*), and the biomass of the stock at the onset of rebuilding relative to the biomass needed to produce MSY (*B_ratio_*). 
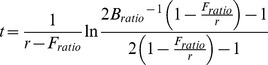



**Figure 1:**
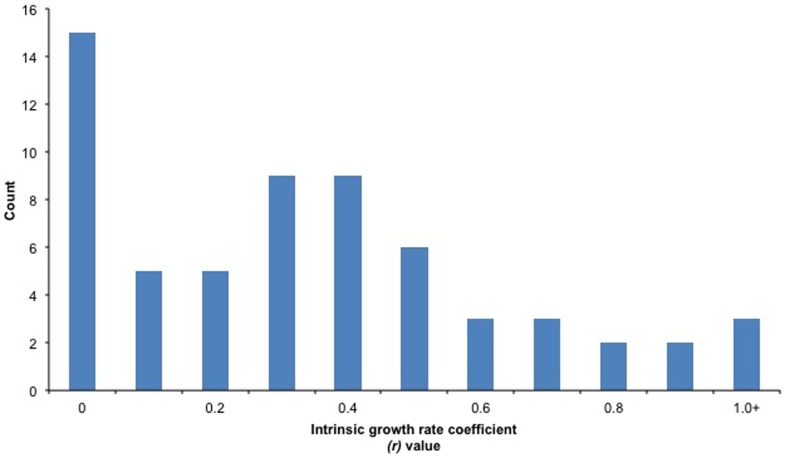
The distribution of intrinsic growth rate coefficients (*r*) for U.S. fish stocks, based on 62 stocks for which F_msy_ or U_msy_ values were available. Note that *r* values labeled 0 on the *x*-axis actually represent 0.01 to 0.09 values.

Safina et al. [9] did not explicitly state what *r* values were used to describe “most stocks”, but they did illustrate that *r* values ranged from 0.1 to 1.5 for 242 fish populations based on the work of Myers et al. [22], [23], with the highest counts occurring between 0.4 and 0.6, forming a bell-shaped curve. Jensen et al. [24] recently reviewed 170 populations of fish and found a similar range of *r* values (0.1 to 1.3), but the distribution was highly skewed toward the left, with 0.1 having the highest counts. Given the disparities in *r* distributions between these two studies, we created *r* distributions specifically for U.S. fish populations, by reviewing 154 stock assessments conducted between 2000 and 2012 (Table S1). Intrinsic rate of increase estimates could be coarsely calculated for 62 of those stocks by doubling the reported *F_msy_* (or the harvest rate at MSY; *U_msy_*) value based on the logistic model relationship of *F_msy_*  =  *r*/2 [25]. The other 92 stock assessments we considered only provided proxies of *F_msy_* or *U_msy_*, and in some cases a fishing mortality estimate was lacking. The resulting *r* distribution for U.S. fish populations was essentially a hybrid of the other two studies, which had a bimodal distribution with peaks at 0.05 and 0.35 (Figure 1).

**Figure 2:**
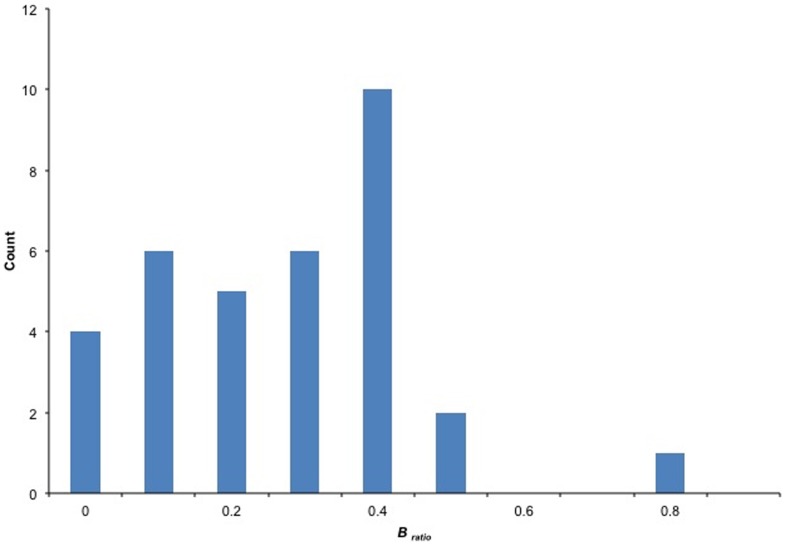
The ratio distribution of biomass at the onset of rebuilding relative to the biomass associated with maximum sustainable yield (*B_ratio_*) for U.S. fish stocks, based on 34 stocks that were determined to be overfished based on the most current stock assessment. Note that *B_ratio_* values labeled 0 on the *x*-axis actually represent 0.01 to 0.09 values.

Similarly, Safina et al. [9] did not discuss what *B_ratio_* values were used to describe “most stocks” in a rebuilding plan. In the United States most stocks are declared overfished when *B_ratio_*_s_ fall below ½ *B_msy_*, although several stocks have more conservative overfished thresholds (e.g., (1-*M*)*B_msy_), where *M* is the natural mortality rate). Rather than use ½ *B_msy_* as *B_ratio_* in our analysis, we reviewed 41 stocks that were in rebuilding plans and documented the biomass of the stock when it was declared overfished and related overfished threshold definition to determine the distribution of *B_ratios_* of U.S. rebuilding stocks (Table S2). The most current stock assessments were used, because they are considered the best available scientific information, which required eliminating seven stocks from consideration because biomasses never dropped below the overfished threshold according to the newest assessments, a result not uncommon when biomass uncertainty across assessments is large [8], [26], [27]. Of the remaining 34 stocks, the current *B_ratio_* distribution for U.S. fish populations ranged from 0.01 to 0.82 (Figure 2).

Considering measures of central tendency across these stocks, the average U.S. fish stock had an *r* value of 0.40 and the average U.S. overfished stock had a *B_ratio_* value of 0.32 at the onset of rebuilding (Table 1; median values were not that different 0.37 and 0.34, respectively). Taking these measures of central tendency and using the Graham-Schaefer model, rebuilding is predicted to occur for the average overfished stock within 4.1 years when *F* = 0. Thus, Safina et al. [9] statement that “most” stocks can rebuild within 5 years appears to be true.

**Table 1:** A comparison of *F_ratios_* (maximum allowed *F_rebuild_*/*F_msy_*), given various intrinsic growth rate coefficients (*r*) values, an initial biomass ratio (*B_ratio_*) of 0.32, and a rebuilding time (*t*) of 10 years.

*r*	*B_ratio_*	*t (yrs)*	*F_ratio_*	10 year exemption
0.050	0.32	10	0.00	Yes
0.100	0.32	10	0.00	Yes
0.200	0.32	10	0.00	Yes
0.300	0.32	10	0.23	No
0.400	0.32	10	0.76	No
0.500	0.32	10	0.85	No
0.600	0.32	10	0.91	No
0.700	0.32	10	0.94	No
0.800	0.32	10	0.97	No
0.900	0.32	10	0.98	No
1.000	0.32	10	0.99	No

Stocks with an *r* value of 0.200 or less are exempt from the mandated 10 year rebuilding requirement, while other stocks are subject to *F_ratios_* ranging from 0.23 to 0.99. Under normal circumstances, the harvest policy for healthy U.S. fish stocks (i.e., not overfished) often ranges between 75% and 95% of *F_msy_* (Carmichael and Fenske 2010).

It is also important to note that these rebuilding times are based on a Graham-Schaefer model that assumes constant conditions of productivity. In reality, marine environments are not constant and the productivity of stocks can be sporadic. Thus, in many cases rebuilding timelines are based on more complex models that use age structured stochastic rebuilding dynamics that better reflect the highly variable nature of recruitment events and uncertainty in the marine environment [28], [29], [30]. Stochastic models generally provide more precautious estimates of population growth compared to the deterministic biomass models used here [31], [32]. Therefore, the deterministic rebuilding timelines presented here are likely to be more optimistic, resulting in shorter rebuilding times.

## How Many Stocks Are Susceptible to a 10-Year Moratorium?

Although no stocks have been subject to a 10-year moratorium, the threat of such a scenario is a major talking point for opponents of the 10-year rebuilding requirement. Sewell et al. [17] recently summarized the rebuilding timelines of 44 U.S. fish stocks, and showed that 23 (52%) of the stocks had 10-year rebuilding timelines. However, none of these stocks were subject to a 10-year moratorium. Instead, the high percentage of 10-year rebuilding plans is the result of managers choosing to set their *T_target_* to the maximum allowed under the SFA, because the calculation of *T_min_* was something less than 10 years. However, this is not to say that some of these stocks may have dramatically reduced fishing mortality rates in order to achieve the 10-year rebuilding timeline (Table 1). For example, the Southern New England/Mid-Atlantic winter flounder stock is in a 10-year rebuilding plan and has a *F_ratio_* (i.e., maximum *F_rebuild_*/*F_msy_*) that is ∼25% of *F_msy_* [8], whereas the normal harvest policy for this groundfish stock is 75% of *F_msy_* [33].

To determine how many U.S. stocks could qualify for a 10-year moratorium, we used the Graham-Schaefer model to identify combinations of *r* and *B_ratio_* values that trigger 10-year moratoriums, where *F* was equal to zero. Our analysis revealed stocks with *r* values ranging from 0.11 to 0.53 and *B_ratio_* values ranging from 0.01 to 0.49 would trigger the 10-year moratorium. Of the 62 stocks for which we have *r* value estimates, half fell in this range, though none of those stocks to date have been subjected to a 10-year moratorium. The lack of 10-year fishing moratoriums suggests that the likelihood of the right conditions occurring (i.e., *r* and *B_ratio_* values that result in a 10-year moratorium) is either not high or that managers are capable of avoiding the moratorium via rebuilding scenarios where *T_min_* is slightly higher than 10 years and thus set *T_max_* higher.

## Does the Use of Generation Time Result in Equitable *F_ratios_* among Rebuilding Stocks?

Historically, the use of the generation time in calculating *T_max_* has not typically been a point of contention in terms of its applicability to rebuilding guidance, although how it is defined can vary. For example, Safina et al. [9] note that the mean generation time for an unfished stock may be much longer than for an overfished stock that has a highly truncated age distribution.

However, we found it interesting that in the past, stakeholders have not questioned why this particular life history characteristic was used in the 1998 guidance. NMFS guidance only notes that it places a reasonable, species-specific cap on the maximum time to rebuild [12], [15], [34]. Although the logarithmic inverse relationship between *r* and generation time is well documented in the scientific literature [35], its utility for scaling *T_max_* with the productivity of the stock has not been empirically investigated to our knowledge.

To evaluate the relationship between *r* values and generation time, we used the database of 154 U.S. stocks that have stock assessments (described earlier), of which 62 stocks had *r* values calculated. Generation times for these 62 corresponding stocks were produced using the “life history tool” found within FishBase [13]. In FishBase, generation time is derived from relationships in optimum age (*t_opt_*), age at length zero (*t_0_*), optimum length, infinity length (*L_inf_*), and the von Bertlanffy growth function (*K*) [13]. Although the accuracy of generation time outputs in FishBase has not been investigated, Thorson et al. [36] recently estimated biases of age at maturity (*t_mat_*) outputs in FishBase, which was found to be relatively accurate and relies on some of the same input parameters as generation time (i.e., *L_inf_, K, and t_0_*). Additionally, the generation times reported in 21 U.S. rebuilding plans and their related *r* values were available for comparison (Table S1); how these generation times were calculated were not provided but we understand that proxies are often used.

**Figure 3:**
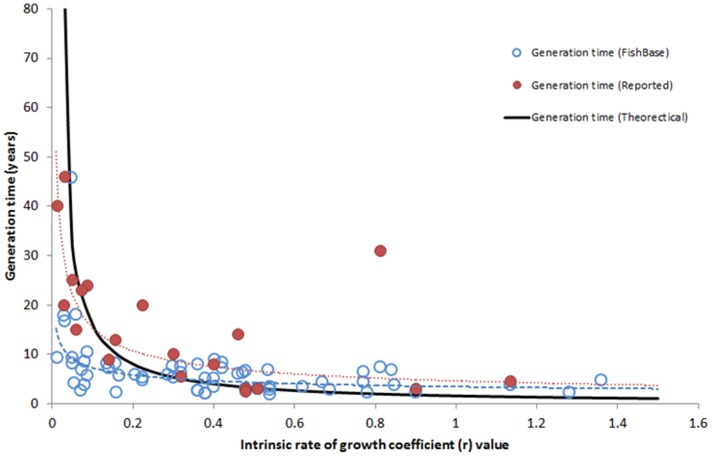
The empirical relationship between intrinsic rate of growth coefficient (*r*) and generation time, for which *r* values (based on *F_msy_* and *U_msy_*) and generation time (based on FishBase and reported values from rebuilding plans) are available. The theoretical relationship between *r* and generation time (*G*) is provided by the equation *G* =  ln*R_0_*/*r*, where *R_0_* (the net reproductive rate per generation) is assumed to be 5, which is roughly the median value of *R_0_* for the *r* and either FishBase or reported generation times.

The logarithmic inverse relationship between *r* values and generation times of stocks is shown in Figure 3. In general, the fit of data is relatively poor (FishBase *R^2^* = 0.31 – dashed line; Reported values *R^2^* = 0.52 – dotted line), but more importantly the contrast between *r* and generation time is lost when *r* values are greater than 0.20. This lack of contrast between *r* and generation time means that stocks with an *r* value of 0.20 or greater will have very similar generation times (i.e., ∼5 years), which disproportionally affects the calculation of *T_max_* and related *F_ratio_* of the stock. For example, using the Graham-Schaefer model and NMFS rebuilding guidance, the average overfished stock (*r* = 0.40 and *B_ratio_* = 0.32) that has a generation time of 5.0 years, a *T_min_* of 4.1 years, a *T_max_* of 9.1 years, and an allowable *F_ratio_* that is 71% of *F_MSY_*. Whereas the *F_ratio_* for a stock with the same generation time (5.0 years) and *B_ratio_* (0.32), but a lower *r* value (0.10) is only 31% of *F_MSY_* (*T_min_* = 16.6 years and *T_max_* = 21.6 years), a 56% reduction compared to the example above.

Although our observations are consistent with the expected inverse relationships between *r* and generation time, these results are based on non-validated data from FishBase and reported generation times for which the methodology used to calculate the values is unknown. Therefore the poor relationships observed here could be the result of various proxies being used to calculated generation time, as opposed to more reliable methods [34]. Regardless, it appears that the generation time lacks the contrasts to be useful scalar of productivity for the majority of U.S. fish stocks and its use likely results in disproportional estimates of *T_max_* and *F_ratio_*.

## Does Overfishing Still Threaten the Success of Rebuilding Plans?

**Figure 4:**
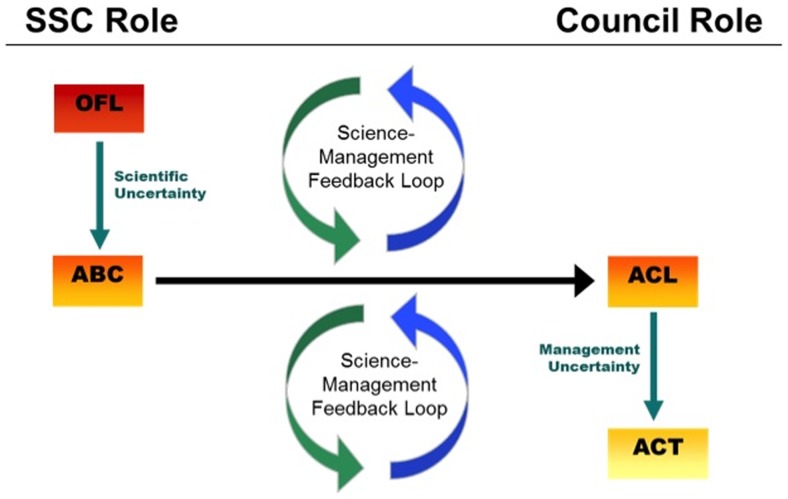
The annual catch limit (ACL) framework, describing how the acceptable biological catch level (ABC) is reduced from the overfishing limit (OFL) based on scientific uncertainty in the estimate of OFL, how the ACL can be set at or below ABC, and how an annual catch target (ACT) can be set below the ACL to account for management uncertainty. The Scientific and Statistical Committee of a Regional Fishery Management Council sets an OFL and ABC, while the Regional Fishery Management Council sets the ACL and ACT; each decision is based on a science-management feedback loop.

In the past, several researchers have shown that overfishing during the initial phases of a rebuilding plan or chronic overfishing of a stock throughout the rebuilding plan were the primary causes for a stock not to rebuild [8], [14], [16], [17], [18]. For stocks that could rebuild in 10 years, the 10-year rebuilding mandate was effective in that it placed a backstop on the time allowed to rebuild, and made preventing overfishing a priority because otherwise drastic cuts to fishing effort near the end of the rebuilding timeline may be needed to meet the 10-year maximum [9]. However, much has changed since 1996 in the way U.S. fisheries are managed. In 2006, the MSA was reauthorized (MSRA) and required, among other things, the use of annual catch limits and accountability measures to prevent overfishing of all stocks within a fishery management plan [14], [15]. These new requirements were implemented through national guidance in 2009 [5], and created an annual catch limit framework that accounts for the scientific and management uncertainty in fisheries management by setting precautionary catch limits (i.e., annual catch limits) below the amount corresponding to maximum sustainable yield (i.e., overfishing limit) (Figure 4). When annual catch limits are exceeded, accountability measures are triggered to correct for the overage and to help prevent chronic overfishing [37]. Annual catch limits and accountability measures are also applied to rebuilding plans, to ensure rebuilding catch limits are not exceeded.

**Figure 5:**
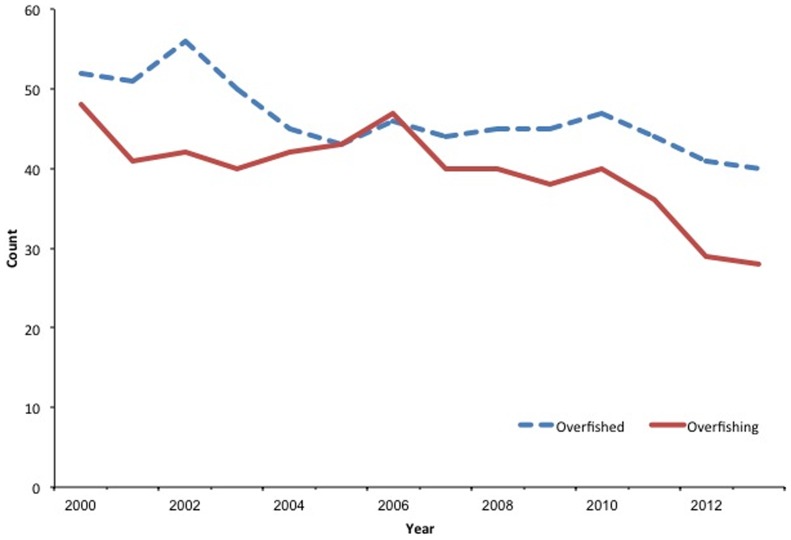
Between 2000 and 2013, overfishing determinations declined from 48 to 28 (a 41% reduction) while overfished determinations declined from 52 to 40 (a 23% reduction).

Given the reauthorized MSA, we evaluated the performance of fisheries management in preventing overfishing by reviewing the *Status of U.S. Fisheries Report to Congress* [38]. These reports summarize the number of stocks determined to be undergoing overfishing or in an overfished state between 2000 and 2013 (at time of writing 2013 data are based on second quarter reports) [38]. During this period, the number of stocks undergoing overfishing declined from 48 to 28 (a 41% decrease) while stocks in an overfished state declined from 52 to 40 (a 23% decrease) (Figure 5). Of the stocks with a known status in 2013, only 13% (26 of 194) were undergoing overfishing and 21% (37 of 175) were overfished. The percentage of stocks undergoing overfishing and stocks in an overfished condition are likely to continue to decrease given that annual catch limits and accountability measures were not fully implemented until 2012 by the U.S. regional fishery management councils. Preliminary data on annual catch limit performance in 2011 and 2012 suggest that approximately 10% of the stocks exceeded their annual catch limit and 7% exceeded the overfishing limit [39]. Therefore it appears that the reauthorized Magnuson-Stevens Act and annual catch limit framework have been successful at limiting (with the intention of ending) overfishing and have added effective provisions that were not in place when the 10-year rebuilding requirement was initially implemented.

The rebuilding framework developed to implement the 10-year requirement, however, has been a very useful concept. Prior to the SFA and related 1998 rebuilding guidance, the only guidance provided for rebuilding plans was that a program must be established for rebuilding the stock over a period of time specified by the Regional Fishery Management Council and acceptable to the Secretary of Commerce [40]. As a result, the rationales for how timelines for rebuilding were chosen were not as transparent as they have been under the original 1998 or revised 2009 guidelines. With the 1998 rebuilding guidance, the *T_min_*, *T_target_*, and *T_max_* framework was created and essentially established lower and upper limits on the allowable time to rebuild, which set the stage for discussing the socio-economic trade-offs in setting the *T_target_* somewhere between *T_min_* and *T_max_*.

The rebuilding framework is also good for developing a roadmap to recovery, because it necessitates the development of long-term projection models to predict how the stock will respond to the fishing mortality rate associated with *T_target_* (*F_rebuild_*). Managers can then use updated data and assessments and subsequent projection models to determine whether they are making adequate progress (i.e., more or less on schedule). If adequate progress is not being made, managers can then evaluate whether the lack of progress is due to inadequate management control (i.e., *F*> *F_rebuild_*), unfounded assumptions or other needed updates to the projection model, or identifiable environmental conditions that changed the expectation of recruitment to the fishery [40], [41], [42], [43], [44]. Depending on the factors identified, managers have an array of management tools they can consider to resolve the underlying issue.

## Alternative Approaches to Calculating *T_max_* Using Fishing Mortality

Given the pros and cons of current rebuilding mandates and related guidance, we recommend two alternative approaches to developing rebuilding timelines. Both approaches rely on constant fishing mortality rates to calculate *T_max_* and avoid discrepancies in how *T_max_* is calculated between short- and long-lived species in terms of *F_ratios_* and the 10-year time limit. Both approaches also fit within the existing rebuilding guidance framework, where *T_min_* and *T_max_* are calculated to define the minimum and maximum times to rebuild, while *T_target_* is still set somewhere in between the two reference points to rebuild in as short a time as possible while taking into account the needs of fishing communities and interactions within the marine ecosystem. However, given the uncertainty in stock assessment projections, the rebuilding framework would only be used for planning purposes, and less for delineating or defining hard deadlines to rebuild. Instead, strong accountability measures (e.g., in-season closure authority, payback provisions, annual catch targets, etc.) could be used to reduce the effects of implementation error and ensure that rebuilding occurs in a timely manner. Such an approach is also recommended in the recent NRC report on U.S. rebuilding plans [8], noting that rebuilding plans that focus more on meeting selected fishing mortality targets than on exact schedules for attaining biomass targets may be more robust to assessment uncertainties, natural variability, and ecosystem consideration, and may have lower social and economic impacts.

The key to these alternative approaches is identifying an acceptable *F* that both meets the existing 10-year rebuilding mandate for the average overfished stock, and is acceptably high to support the fishery during rebuilding. As noted earlier, the average overfished stock (*r* = 0.40 and *B_ratio_* = 0.36) can rebuild in 4.1 years (roughly 5 years) when the fishing mortality is set to zero and productivity is constant, and this was presumably the rationale used for creating the 10-year rebuilding timeframe (i.e., twice the time to rebuild for “most” stocks). Thus, one alternative to calculating *T_max_* is to simply multiply *T_min_* by two (*T_max_* = 2 * *T_min_*) to allow the stock twice the time to rebuild. Using this alternative approach means the average overfished stock would be expected to rebuild in 8.2 years, and the maximum allowable *F_ratio_* for the stock would be 66% of *F_msy_*. Furthermore, the *F_ratio_* of 0.66 is constant among different *r* types of stocks, because *F_msy_* is a function of *r* (*F_msy_* = *r*/2; 25). Lastly, it is worth noting that New Zealand's Ministry of Primary Industry uses this approach for calculating *T_max_* and it was highlighted by the NRC report on U.S. rebuilding plans as a model to consider [8], [45].

**Figure 6:**
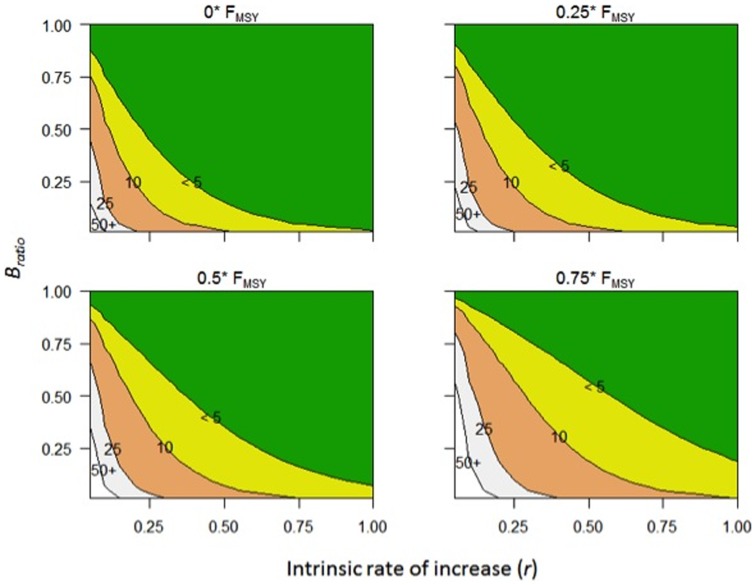
Rebuilding times (*t*) with various *F_ratios_* (0%, 25%, 50%, and 75%), using the Graham-Schaefer model. Rebuilding time depends only on the intrinsic rate of increase (*r*), fishing mortality relative to *F_msy_* (*F_ratio_*), and the biomass at the onset of rebuilding relative to *B_msy_* (*B_ratio_*). The average overfished stock (*r* = 0.40 and *B_ratio_* = 0.32) can rebuild in 4.1 years with *F_ratio_* = 0.0, or 10.0 years when *F_ratio_* = 0.76.

The second alternative tries to rectify the difference between a *T_max_* that is 8.2 years (based on *T_min_* * 2) and the 10 years allowed for the average overfished stock. We ran the Graham-Schaefer model with different F*_ratios_* (assuming *T_target_* equals *T_max_*) until the time to rebuild (*t*) equaled 10 years. Our analysis revealed that the average overfished stock could rebuild in 10 years (*T_max_*) using a *F_ratio_* that was 76% of *F_msy_* (Figure 6). Though the use of surplus production models admittedly may produce more optimistic results than stochastic age structured models, the *F_ratio_* of 76% of *F_msy_* coincidentally aligns with the 75% *F_msy_* harvest control rule that is commonly used in U.S. fisheries [33], [46]. The 75% *F_msy_* harvest control rule gained popularity in the 1990s as a precautionary approach to fisheries management, when studies revealed that such a rule reduces the chances of overfishing, results in equilibrium yields of 94% of MSY or higher, and equilibrium biomass levels between 125% and 131% *B_msy_*—a relatively small sacrifice in yield for a relatively large gain in biomass [34], [47], [48]. Additionally, NMFS national guidance on rebuilding overfished stocks notes that if a stock has not rebuilt by *T_max_*, then the fishing mortality rate should be maintained at *F_rebuild_* or 75% *F_msy_*, whichever is less [15]. Given the similarities to the 75% harvest control rule and current rebuilding guidance, we re-ran the analysis using 75% as the *F_ratio_* and found that the average overfished stock could rebuild in 9.8 years. Since there is essentially no difference in the rebuilding time (i.e., 9.8 vs. 10.0), we will refer to this alternative approach as the 75% *F_msy_* rebuilding approach.

The intent of our analyses was to demonstrate that a *T_max_* approach based on a constant *F* could meet the 10-year rebuilding requirement, and result in a more consistent and simplified rebuilding strategy. Assuming the history behind the 10 year requirement reported in Safina et al. [9] was accurate, our two approaches to defining *T_max_* (i.e., *T_min_* * 2 and 75%*F_msy_*), along with ACL and AM management and using *T_max_* as a planning tool rather than a hard deadline, address the majority of stakeholders concerns with the current rebuilding framework including: (1) most stocks (i.e., over 50%) can still rebuild within 10 years, (2) rebuilding stocks would no longer be susceptible to a10-year moratorium; (3) there would be no discontinuities in allowable *F_ratios_* among short- and long-lived species due to the use of 1+ generation time calculations; and (4) managers could focus on controlling *F_rebuild_*, as opposed to trying to control the biomass of the stock which is harder to estimate and can be driven by environmental conditions. Regarding this later point, evaluating the biomass of the stock is still needed to determine whether the rebuilding target (i.e., *B_msy_*) has been reached or not.

Our suggested approaches, however, are limited by the current *T_min_*, *T_target_*, *T_max_* framework recommended in NMFS national guidance on rebuilding overfished stocks that operationalizes the rebuilding mandates of the MSA. Alternatives to this framework could be considered, if these mandates were revised. For example, the NRC report on U.S. rebuilding encouraged the use of harvest control rules that reduce fishing mortality as the biomass of the stock declines [8]. Harvest control rules constructed in such a manner would likely prevent stocks from ever being declared overfished in the first place, as more conservative fishing mortality rates are applied as the stock declines [8], [49], [50]. These types of harvest control rules can also be constructed in such a way that there are tiered categories of management action, where severely depleted stocks may be subject moratorium until the biomass of the stock reaches a minimum threshold (e.g., ¼ *B_msy_*), whereby a more structured *F*-based approach is applied until the stock rebuilds to its target level of biomass [45].

Lastly, while we have only focused on trying to resolve the 10-year rebuilding dilemma of the current rebuilding mandates and guidelines, there is much more analysis that goes into minimizing the economic, social, and ecological impacts of rebuilding a fishery. There is also a myriad of other management tools that managers can use to rebuild fish stocks, such as the use of marine protected areas [8], [51], [52], buy-back programs to reduce fishing capacity [53], [54], stock enhancement [55], and habitat restoration [56], [57]. The most effective rebuilding plans will likely take a portfolio approach to rebuilding stocks, by deploying a wide array of management tools, rather than solely relying on controlling the fishing mortality or the biomass of the stock.

## Supporting Information

Table S1 **A list of 154 assessed stocks used in the analysis related to estimates of intrinsic rate of growth (r), generation time, and age at first maturity.**
(XLSX)

Table S2 **A list of the 34 U.S. fish stocks in rebuilding plans for which the biomass at the onset of rebuilding (B_ratio_) was reported.**
(XLSX)
